# ﻿Ultrastructure of three species of *Surirella* (Bacillariophyta) from Lake Qinghai, China, with descriptions of two new species

**DOI:** 10.3897/phytokeys.263.162632

**Published:** 2025-09-26

**Authors:** Ya-Lun Ma, Qiao-Mu Peng, Patrick Rioual, Bing Liu, Ji-Yan Long, Bin Yang

**Affiliations:** 1 College of Biology and Environmental Sciences, Jishou University, Jishou 416000, China Jishou University Jishou China; 2 Key Laboratory of Cenozoic Geology and Environment, Institute of Geology and Geophysics, Chinese Academy of Sciences, P.O. box 9825, Beijing 100029, China Institute of Geology and Geophysics, Chinese Academy of Sciences Beijing China; 3 CAS Center for Excellence in Life and Paleoenvironment, Beijing 100044, China CAS Center for Excellence in Life and Paleoenvironment Beijing China; 4 School of Life Sciences, Nanjing Normal University, Nanjing 210000, China Nanjing Normal University Nanjing China

**Keywords:** Brackish water, canal raphe, diatoms, fibula, marginal depression, portula

## Abstract

Three *Surirella* species found at the same locality in Lake Qinghai, northwestern China, are studied using light and scanning electron microscope observations. Two species are proposed as new to science and named *S. ectorii***sp. nov.** and *S. qinghainensis***sp. nov.** The third species is identified as *S. brightwellii* W. Smith. Both *S. brightwellii* and *S. qinghainensis* have valves with an ovate outline and a broadly rounded headpole and cuneate footpole, whereas *S. ectorii* has valves with a nearly elliptical outline and an almost isopolar headpole and footpole. Both *S. ectorii* and *S. qinghainensis* produce a marginal row of costae on the wall of the raphe canal, whereas *S. brightwellii* does not possess this feature. This study provides further insights into the diversity of endemic diatom species inhabiting the ancient Lake Qinghai and illustrates the variability of valve ultrastructure in the genus *Surirella*.

## ﻿Introduction

Morphological and molecular analyses have led to the new circumscription of the diatom genus *Surirella* Turpin, and the synapomorphy for *Surirella* was proposed by Ruck et al. (2016): “communication between the raphe canal and cell interior is through simple portulae created by fibulae of various thickness.” According to this synapomorphy, the newly defined genus *Surirella* includes *S. striatula* Turpin, *S. sella* Hustedt, the Pinnatae section of Surirella, and *Cymatopleura* W. Smith (Ruck et al. 2016). This new definition of *Surirella* by Ruck et al. (2016) has generally been accepted by diatom researchers, and a few new diatom species that previously would have been ascribed to the old genus *Cymatopleura* (now merged into *Surirella*) have been described as new species of *Surirella* (Liu et al. 2019, 2021). In addition, some species of *Cymatopleura* were transferred to the genus *Surirella* (Liu et al. 2021; Van de Vijver et al. 2024). Furthermore, new species belonging to the group of *SurirellaPinnatae* have also been published since 2016 (e.g., Long et al. 2022, 2023; Solak et al. 2023). The ultrastructure and types of both fibulae and portulae were clearly illustrated in the above papers, and these studies contributed to improving our understanding of the synapomorphy of *Surirella*.

Species of the genus *Surirella* have been found to live in freshwater, brackish, and fully marine habitats. The recently described new species of *Surirella* from China were all found in freshwater (Liu et al. 2019, 2021; Long et al. 2022, 2023), and no new brackish-water or marine species of *Surirella* have been reported recently. Almost 40 years ago, Krammer & Lange-Bertalot (1987) observed and discussed some brackish to marine species of *Surirella*, such as *S. brebissonii* Krammer & Lange-Bertalot, *S. brightwellii* W. Smith, *S. crumena* Brébisson ex Kützing, and *S. peisonis* Pantocsek, but did not provide detailed descriptions of the valve ultrastructure for these species. Since that work by Krammer and Lange-Bertalot (1987), very few SEM images of these taxa have been published in the literature. Wojtal (2013) showed the external structure of a couple of valves of *S. brightwellii*. Stenger-Kovács and Lengyel (2015) provided SEM images for the external and internal views of *S. peisonis*. The fine structures, however, were not discussed in these publications. We did not find any recently published SEM images for *S. crumena*. This lack of detailed description makes the correct identification of these taxa still very difficult for diatom researchers.

Lake Qinghai is the largest inland brackish-water lake in China. The earliest diatom record of Lake Qinghai comes from a report by the Lanzhou Institute of Geology (1979), which listed 18 diatom genera but provided no species names or illustrations. Later studies gradually expanded this work. For example, Peng et al. (2013) reported seasonal diatom assemblages in trap samples, identifying 56 diatom species from 32 genera (mainly brackish and marine taxa), but also without illustrations. Since 2014, researchers have discovered, published, and clearly illustrated seven new diatom species, one new variety, one new record for China, and three known species (Table 1). Peng et al. (2013) concluded that Lake Qinghai’s diatoms are mainly brackish and marine taxa, a finding confirmed by later studies of genera such as *Hippodonta* Lange-Bertalot, Witkowski & Metzeltin, *Gyrosigma* Hassall, *Ctenophora* (Grunow) D. M. Williams & Round, *Berkeleya* Greville, and *Entomoneis* Ehrenberg. An exception is *Diatoma sinensis* Bing Liu & Rioual, which belongs to a genus typically considered freshwater (Round et al. 1990) yet was found in the saline environment of Lake Qinghai. In this study, we observed and described three *Surirella* species found in Lake Qinghai and contributed new knowledge of the diatom flora of Lake Qinghai.

**Table 1.** Fifteen diatom species found in Lake Qinghai since 2014, including newly described species and the first record in that lake or for China.

Number	Scientific names	Taxon status and reference
1	*Hippodonta qinghainensis* Peng & Rioual	New species, Peng et al. 2014
2	Gyrosigma peisonis var. majus Peng, Rioual & Sterrenburg	New variety, Peng et al. 2016
3	*Ctenophora sinensis* Bing Liu & D.M. Williams	New species, Liu et al. 2020
4	*Berkeleya fennica* Juhlin-Dannfelt	New record for China, Gong et al. 2020
5	*Pinnularia qinghainensis* Bing Liu & S. Blanco	New species, Deng et al. 2021
6	*Entomoneis sinensis* Bing Liu & D.M. Williams	New species, Long et al. 2022
7	*Entomoneis qinghainensis* Bing Liu & D.M. Williams	New species, Long et al. 2022
8	*Entomoneis paludosa* (W. Smith) Reimer	Known species, Long et al. 2022
9	*Diatoma sinensis* Bing Liu & Rioual	New species, Yuan et al. 2022
10	*Tryblionella apiculata* W. Gregory emend. Liu et Rioual	Known species, Ma et al. 2025
11	*Tryblionella levidensis* W. Smith emend. Liu et Rioual	Known species, Ma et al. 2025
12	*Tryblionella qinghainensis* Bing Liu et Rioual	New species, Ma et al. 2025
13	*Surirella brightwellii* W. Smith	Known species, this study
14	*Surirella ectorii* Bing Liu & Rioual	New species, this study
15	*Surirella qinghainensis* Bing Liu & Rioual	New species, this study

## ﻿Materials and methods

### ﻿Site description

Three sampling sites were chosen from the lakeshore waters of Lake Qinghai (see Liu et al. 2020: 116, fig. 1). Geographically, Lake Qinghai is located between longitudes 99°36' and 100°47', and latitudes 36°32' and 37°15' in Qinghai Province, China. It is the largest inland brackish-water lake in China. The lake is expanding rapidly, by ~2.9 km^2^ per year (Ling et al. 2023), and its surface water area is currently 4573 km^2^, while the elevation of its surface is ca. 3200 m asl. Its climate belongs to the plateau continental climate. The average annual temperature is ca. –0.7 °C, and the ranges of the average annual precipitation and the average annual evaporation in the lake region are 319–395 mm and 800–1000 mm, respectively (Luo et al. 2017). More than 50 rivers and streams run into Lake Qinghai, and there is no outlet to discharge the lake water, as Lake Qinghai is hydrologically closed and only loses water to surface evaporation. The lake has an 18.3 m average water depth, and the maximum depth is 26.6 m. The average values for alkalinity and pH are 25.6 mmol L^–1^ and 9.2, respectively (Peng et al. 2014). There is a 3-month period of ice cover (from mid-November to mid-February), so that the growth period for diatoms in Lake Qinghai is mainly from May to October.

### ﻿Sampling

At the three sampling sites in Lake Qinghai (see Liu et al. 2020: 116, fig. 1), there are many submerged stones with a yellow-brown surface, which indicates that abundant diatoms are growing on them. Each stone sampled was placed on a plastic plate, and then its surfaces were brushed using a toothbrush, with the brushed-off diatom samples washed into the plate. The samples were transferred to a 100 mL sampling bottle and fixed with 70% ethanol. Two bottles of diatom sample were collected for each sampling site. Simultaneously with the collection of diatom samples, the temperature, pH, and conductivity of the water were measured in situ with a portable multimeter (HQ40D, HACH Company).

### ﻿Methods

The samples were processed (cleaned of organic material) for microscope examination using 10% HCl and 30% H_2_O_2_. Permanent slides were prepared using the resin Naphrax (Brunel Microscopes Ltd., UK). These slides were examined, and the specimens photographed, using a Leica DM3000 light microscope (**LM**) and a Leica MC190 HD digital camera. The slides with the holotype specimens are deposited in the Herbarium of Jishou University, Hunan, People’s Republic of China (**JIU**).

Samples were further examined using scanning electron microscopy (**SEM**). Several drops of the selected cleaned diatom material were air-dried onto glass coverslips. Coverslips were attached to aluminum stubs using a double-sided conductive carbon strip and sputter-coated with platinum (Cressington Sputter Coater 108auto, Ted Pella, Inc.). Samples were examined and imaged using a field emission scanning electron microscope (**FE-SEM**) Sigma HD (Carl Zeiss Microscopy) available at Huaihua University, China.

Diatom terminology largely follows Round et al. (1990) and Ruck and Kociolek (2004), with terms such as costa-stria bundle (**CSB**) and mantle sinking against fibula (MSAF) taken from Liu et al. (2019). Following Barber and Haworth (1981), we also replaced the term “over-fibula costa” with “over-fibula rib,” because this structure is a rib rather than a costa (thickened rib).

## ﻿Results

### ﻿ Surirella brightwellii

Taxon classificationPlantaeSurirellalesSurirellaceae

W. Smith 1853

FA3806F9-FD15-5338-9FF1-F27382D5F7B5

Figs 1, 2, 3

#### Description.

**Figure 1.**
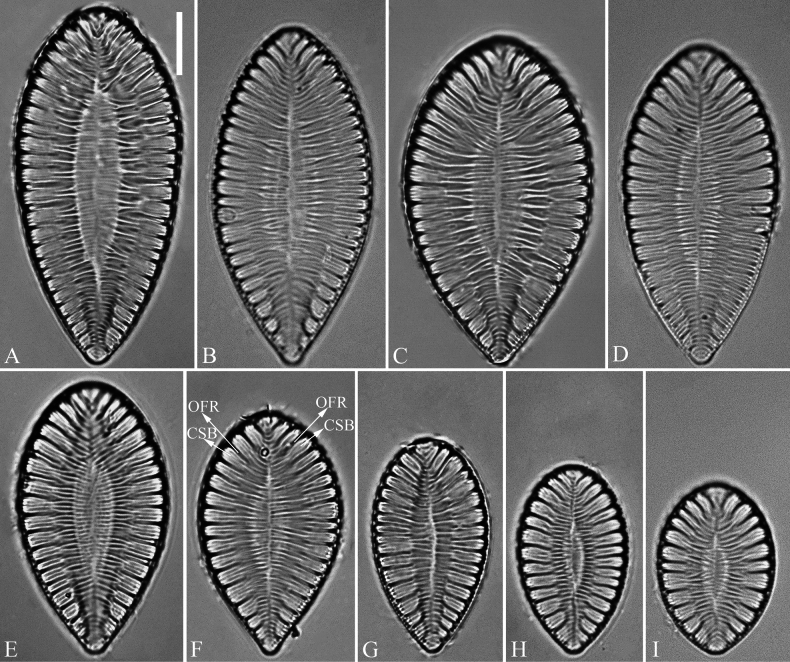
*Surirella brightwellii*, LM, × 1000 A–I. Nine valves showing a size diminution series; note the distinctive costa-stria bundles, over-fibula ribs (CSB and OFR, labelled in F), and some costae reaching the valve midline. Scale bar: 10 μm.

***LM*** (Fig. 1). Valve outlines ovate with broadly rounded headpole and cuneate footpole (Fig. 1). Valve dimensions (n = 102): length range 19–56 μm, width range 13–29 μm at its widest region. Costa-stria bundles (CSBs) distinct, alternating with over-fibula ribs (OFRs) from pole to pole (labelled in Fig. 1F). CSBs parallel at the valve middle, radiate approaching two apices. Fibulae visible, short (judged by OFRs, labelled in Fig. 1F), 5–7 in 10 μm.

**Figure 2.**
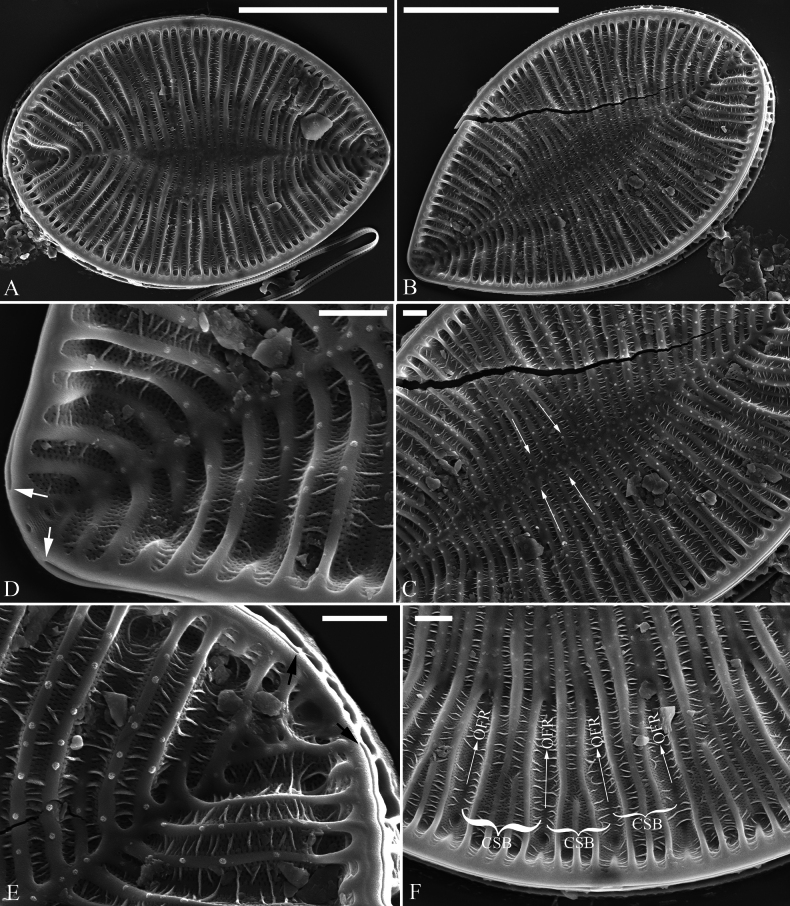
*Surirella brightwellii*, external view, SEM A, B. Two valves; note the distinctive costae; C. Detail of valve middle part from B; note some costae reaching valve midline (arrows); D. Footpole detail from B; note the interrupted distal raphe endings (two white arrows) and slit-like outer openings of areolae; E. Headpole detail from B; note the interrupted distal raphe endings (two black arrows); F. Valve side detail from A showing costa-stria bundles (CSB) and over-fibula ribs (OFR). Scale bar: 10 μm (A, B); 1 μm (C–F).

**Figure 3.**
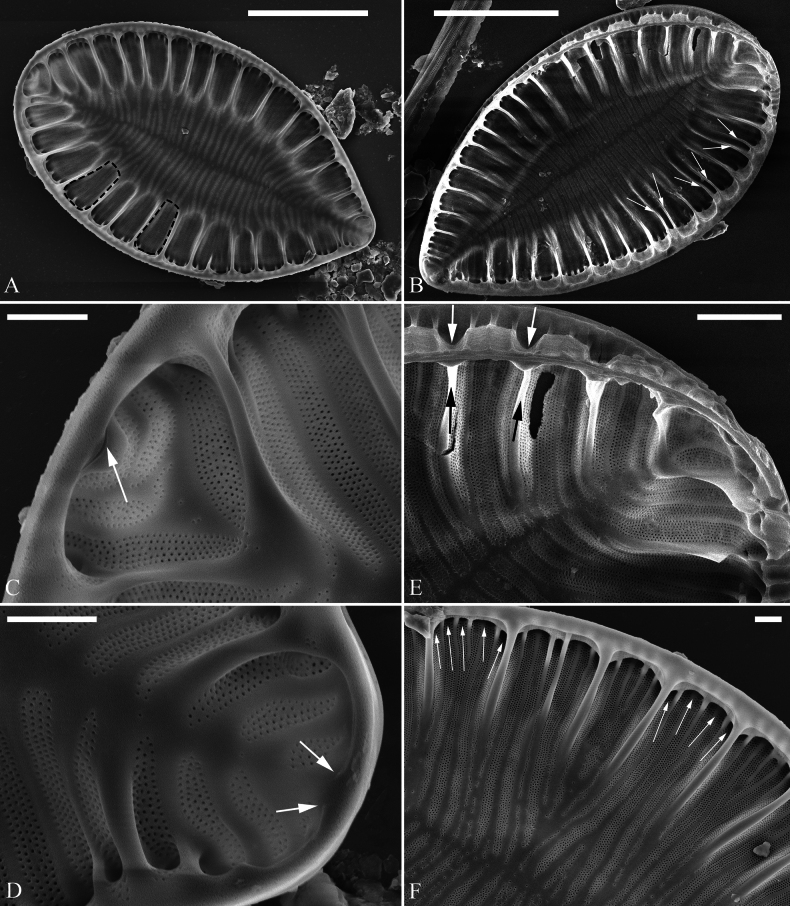
*Surirella brightwellii*, internal view, SEM A, B. Two complete valves; note the slim, short fibulae and marginal trough-like depressions (indicated by black dotted lines in A) and some double fibulae (arrows in B); C. Headpole, detail from A; note the continuous distal raphe (arrow), multiseriate striae and not rimmed inner openings of areolae; D. Footpole from A; note the interrupted distal raphe (two arrows); E. Headpole from B; note the costa-stria bundles and each mantle sinking against a fibula (two white arrows and two black arrows); F. Valve side view showing 2–5 portulae between two adjacent fibulae (arrows). Scale bar: 10 μm (A, B); 1 μm (C–F).

***SEM*** (Figs 2, 3). Externally, raphe canal located directly on the mantle, and wall of raphe canal hyaline (Fig. 2A, B), distal raphe endings straight, interrupted at both headpole and footpole (Fig. 2D, E). Surface costae mostly raised, some reaching valve midline (Fig. 2C). Each CSB composed of ca. 2–4 costae and 3–5 striae (Fig. 2F). Outside openings of areolae slit-like or rounded (Fig. 2D, E). Striae multiseriate, composed of ca. 2–5 rows of areolae (Fig. 2D, E), 19–22 in 10 μm (measured at the valve margin from SEM images, n = 4). Surface siliceous warts and reticulate thickenings produced on costae and between adjacent two costae, respectively (e.g., Fig. 2D, E). Internally, wall of raphe canal not growing conspicuously into cell cavity, leaving portulae visible (Fig. 3A, B). Fibulae slim, short, sometimes doubled (Fig. 3B, arrows), spanning ca. 1/4 of valve width, not extending to valve midline except at two valve poles. Marginal trough-like depressions present around entire raphe canal (Fig. 3A, black dotted lines). Raphe continuous at headpole (Fig. 3C, arrow) whereas interrupted at footpole (Fig. 3D, two arrows). 2–5 portulae produced between two adjacent fibulae (Fig. 3F, arrows). Inner openings of areolae rounded, not rimmed. Each mantle sinking against a fibula (Fig. 3E, arrows).

#### Ecology and distribution.

The type localities of *Surirella brightwellii* are the coasts of Norfolk and Lewes (United Kingdom), and Smith stated that it was a fresh or brackish water species (Smith 1853). According to Krammer and Lange-Bertalot (1987), the syntypes designated by Hoover (1976) originate from a locality in Sussex (a brackish habitat). In this study, *Surirella brightwellii* was found to be a dominant species in the benthic diatom community in Lake Qinghai. At the sampling point where it was collected near the lakeshore of Lake Qinghai (36°50'34"N, 99°42'39"E, 3210 m asl.) on July 19, 2019, the conductivity was 16.3 ± 0.1 mS·cm^–1^, pH was 9.14 ± 0.01, and water temperature was 15.5 ± 0.3 °C. These data support previous findings that *S. brightwellii* is a brackish diatom species.

### ﻿ Surirella ectorii

Taxon classificationPlantaeSurirellalesSurirellaceae

Bing Liu & Rioual sp. nov.

8AFEF91A-8EC3-5508-B711-C7BA141FE7FA

Figs 4, 5, 6

#### Holotype.

**Figure 4.**
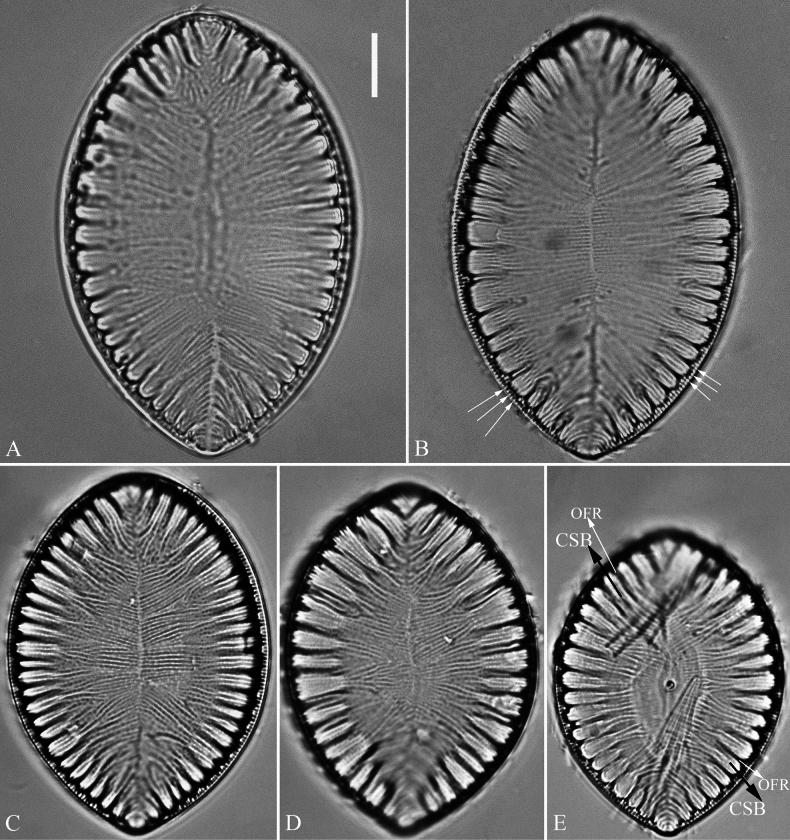
*Surirella ectorii* sp. nov., LM, × 1000 A–E. Five valves showing a size diminution series; note the almost symmetric valve outlines relative to the apical axis, relatively planar valve surfaces, distinctive costa-stria bundles alternated with over-fibula ribs (CSB and OFR, labelled in E), and a marginal row of costae produced on the wall of raphe canal (B, indicated by six arrows); B. Illustration of holotype specimen. Scale bar: 10 μm.

Specimen circled on slide DIA2025001 (= Fig. 4B), deposited in the Herbarium of Jishou University (JIU), China. Registration: http://phycobank.org/105636.

#### Type locality.

China. Qinghai Province, Lake Qinghai, a sampling site near the lakeshore, 36°50'34"N, 99°42'39"E, 3210 m asl., collected by Bing Liu, July 19^th^, 2019.

#### Description.

***LM*** (Fig. 4). Valve outlines nearly elliptical with almost isopolar headpole and footpole. Valve dimensions (n = 24): length range 44–69 μm, width range 34–45 μm at its widest region. Costa-stria bundles (CSBs) distinct, alternating with over-fibula ribs (OFRs) from pole to pole (labelled in Fig. 4E). CSBs radiate throughout the valve surface. Fibulae visible, short (judged by OFRs, labelled in Fig. 4E), 4–6 in 10 μm. A marginal row of costae produced on the wall of raphe canal (e.g., Fig. 4B, arrows), 16–18 in 10 μm.

**Figure 5.**
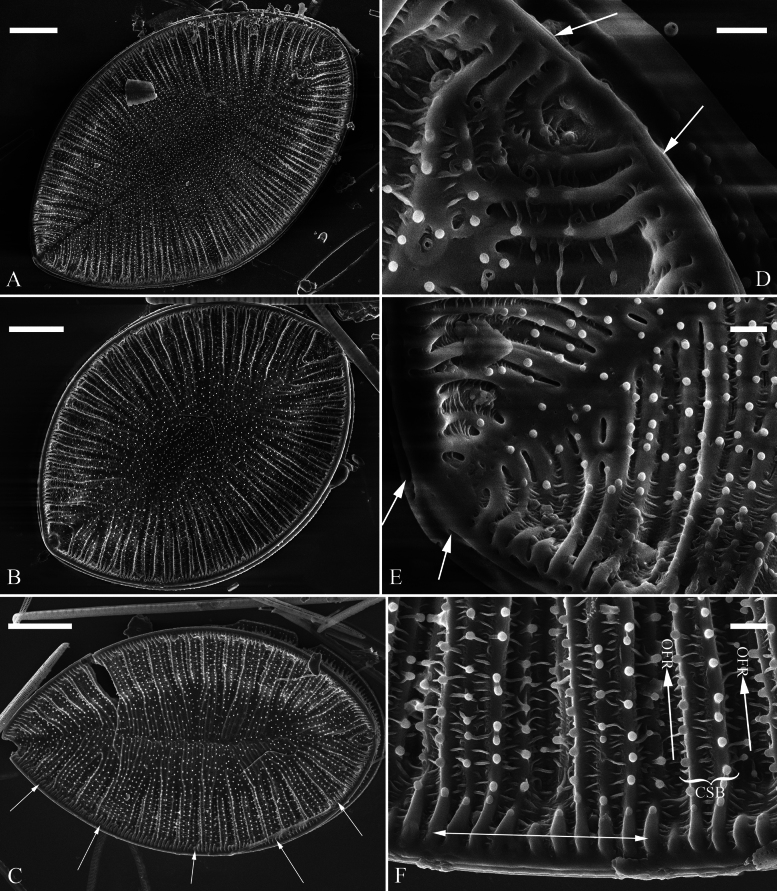
*Surirella ectorii* sp. nov., external view, SEM A–C. Three valves; note the relatively planar valve surfaces, prominent siliceous warts throughout the valve surface, and a marginal row of costae produced on the wall of the canal raphe (C, arrows); D. Headpole detail from A; note the interrupted distal raphe endings (two arrows); E. Footpole detail from A; note the costae, siliceous warts, reticulate thickenings, and interrupted distal raphe endings (two arrows); F. Valve side detail showing the costa-stria bundles (CSB), over-fibula ribs (OFR), and a marginal row of costae produced on the wall of the raphe canal (double-headed arrow). Scale bar: 10 μm (A–C); 2 μm (D–F).

**Figure 6.**
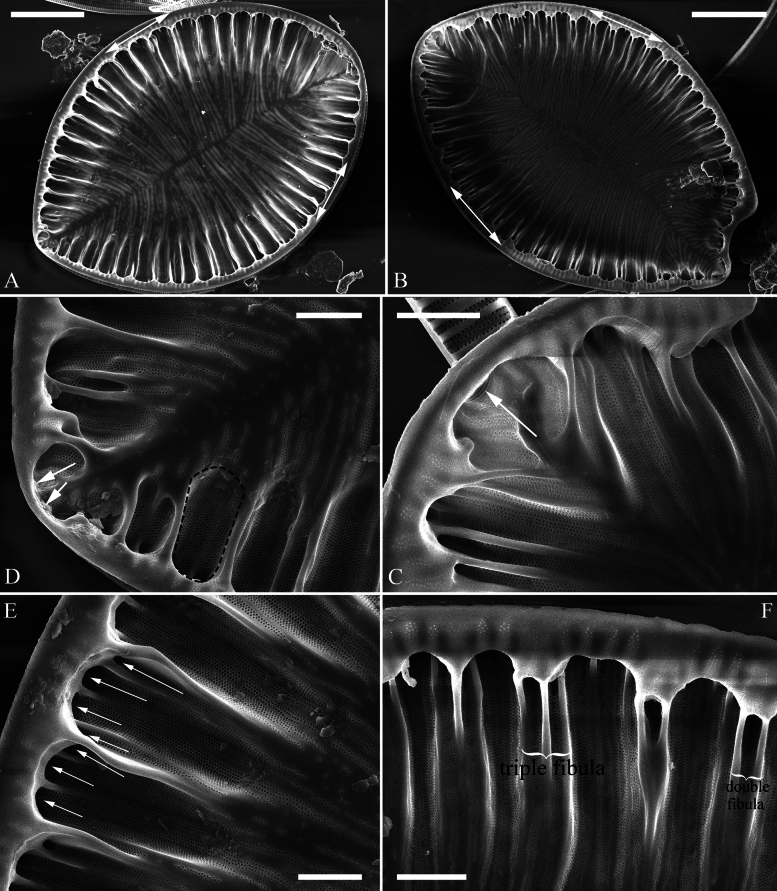
*Surirella ectorii* sp. nov., internal view, SEM A, B. Two valves; note the raphe canal wall grows more into the cell cavity (double-headed arrows) and the slim, short fibulae; C. Headpole detail; note the continuous distal raphe (arrow), multiseriate striae, and not rimmed inner openings of areolae; D. Footpole detail from A; note the interrupted distal raphe (two arrows) and marginal depressions (indicated by the black dotted line); E. Valve side view showing 2–5 portulae between two adjacent fibulae (arrows); F. Another side view showing the double and triple fibula. Scale bar: 10 μm (A, B), 2 μm (C–F).

***SEM*** (Figs 5, 6). Externally, raphe canal located directly on mantle (Fig. 5A–C), and wall of raphe canal bearing a marginal row of costae (Fig. 5C, arrows, Fig. 5F, double-headed arrow). Distal raphe endings straight, interrupted at both headpole and footpole (Fig. 5D, E). Surface costae mostly slightly raised, some reaching valve midline (Fig. 5C). Each CSB often composed of ca. 2–5 costae and 3–6 striae (Fig. 5F). Outside openings of areolae slit-like (Fig. 5F). Striae multiseriate, composed of ca. 2–5 rows of areolae (Figs 5F, 6), 15–19 in 10 μm (measured at the valve margin from SEM images, n = 3). Surface siliceous warts and reticulate thickenings produced on costae and between adjacent two costae, respectively (e.g., Fig. 5D–F). Internally, wall of raphe canal slightly growing into cell cavity, whereas portulae visible (Fig. 6A, B). Fibulae slim, short, sometimes doubled or tripled (Fig. 6F, arrows), spanning ca. 1/4 of valve width, not extending to valve midline except at two poles. Marginal trough-like depressions present around entire raphe canal (Fig. 6D, black dotted line). Raphe continuous at headpole (Fig. 6C, arrow) whereas interrupted at footpole (Fig. 6D, two arrows). 2–5 portulae produced between two adjacent fibulae (Fig. 6F, arrows). Inner openings of areolae rounded, not rimmed.

#### Etymology.

Named after the late diatomist from Luxembourg, Luc Ector (1962–2022), for his dedication to diatom taxonomy and his help with our studies of diatoms in China.

#### Vernacular name.

The Chinese name is “埃氏双菱藻”.

#### Ecology and distribution.

*Surirella ectorii* was commonly found on the sediment at the surface of the stones collected in Lake Qinghai but not in large abundance. In the samples it is found in association with *S. brightwellii*. So far, *S. ectorii* has only been found in the type locality and may therefore be considered as a brackish diatom endemic to Lake Qinghai.

### ﻿ Surirella qinghainensis

Taxon classificationPlantaeSurirellalesSurirellaceae

Bing Liu & Rioual sp. nov.

73E489B8-D4F9-5F6B-96B2-26FD71670FA7

**Figure 7.**
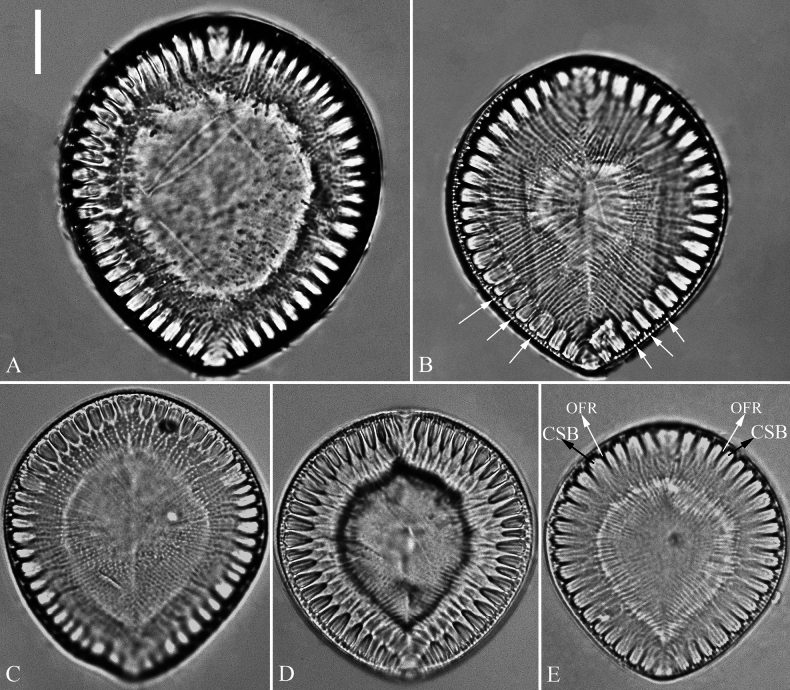
*Surirella qinghainensis* sp. nov., LM, × 1000 A–E. Five valves showing a size diminution series; note the distinctive costa-stria bundles (CSB) alternated with over-fibula ribs (labelled in E) and a marginal row of costae produced on the wall of raphe canal (B, arrows); B. Illustration of holotype specimen. Scale bar: 10 μm.

**Figure 8.**
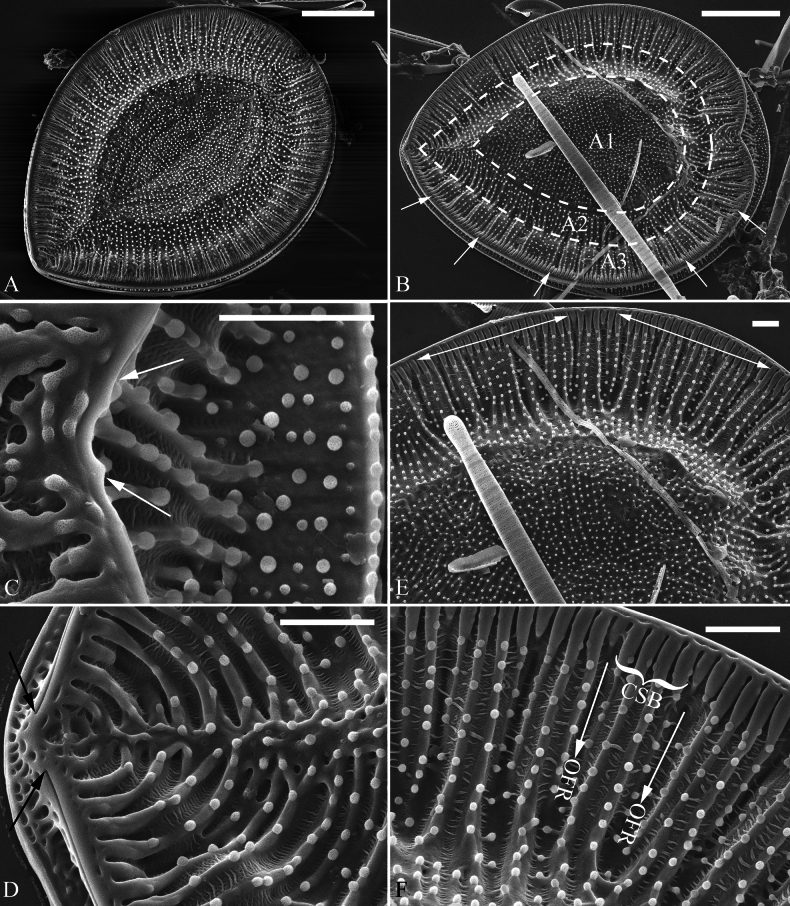
*Surirella qinghainensis* sp. nov., external view, SEM A, B. Two complete valves; note the prominent siliceous warts scattered throughout the valve surface that is differentiated into three distinct areas (labelled A1, A2, and A3 in B) and a marginal row of costae produced on the wall of the raphe canal (five arrows); C. Headpole detail from B; note the interrupted distal raphe endings (two white arrows); D. Footpole detail from B; note the costae, siliceous warts, reticulate thickenings, and interrupted distal raphe endings (two black arrows); E. Valve side detail showing a marginal row of costae (two double-headed arrows); F. Valve side detail showing the costa-stria bundles (CSB) and over-fibula ribs (OFR). Scale bar: 10 μm (A, B); 2 μm (C–F).

**Figure 9.**
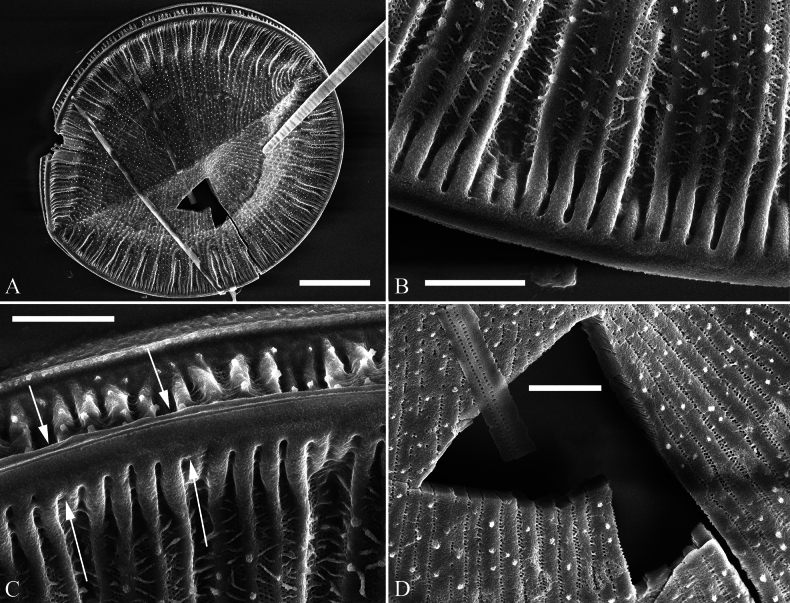
*Surirella qinghainensis* sp. nov., external view, SEM A. A broken valve; B. Valve side detail showing the multiseriate striae and its outer openings; C. Mantle view from A; note each mantle sinking corresponding to each over-fibula rib (arrows); D. A broken part from A showing the strongly silicified valve and uniseriate striae near the valve midline. Scale bar: 10 μm (A); 2 μm (B–D).

**Figure 10.**
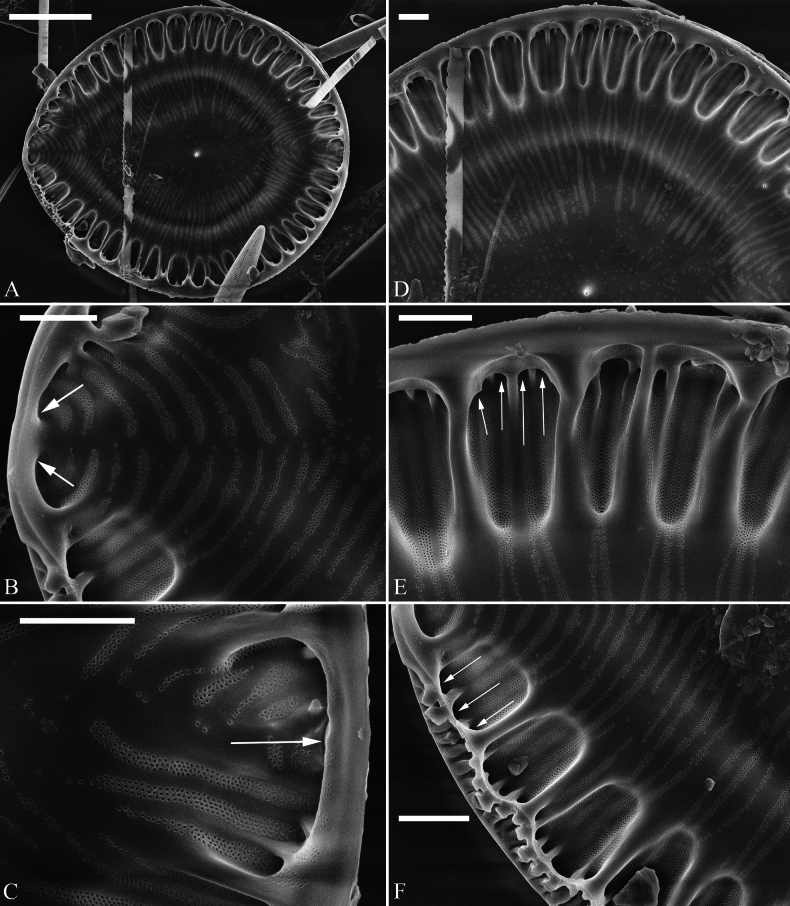
*Surirella qinghainensis* sp. nov., internal view, SEM A. A complete valve; note the short fibulae; B. Footpole from A; note the multiseriate striae, the rimmed inner openings of areolae, and the interrupted distal raphe (two arrows); C. Headpole from A; note the continuous distal raphe (arrow); D. Valve side view showing the distribution of striae from valve margin to center; E, F. Other valve side views showing the marginal depressions and 2–4 portulae between two adjacent fibulae (arrows). Scale bar: 10 μm. (A); 2 μm (B–F).

**Figure 11.**
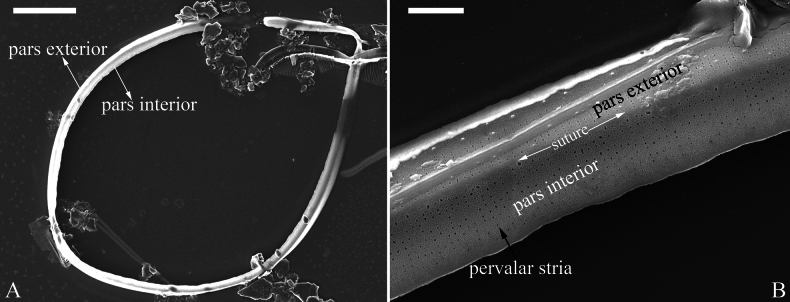
*Surirella qinghainensis* sp. nov., girdle band, SEM A. A girdle band, note its open nature; B. Detail from A showing details of the pars exterior and interior, the suture, and pervalvar striae. Scale bar: 10 μm (A); 1 μm (B).

Figs 7, 8, 9, 10, 11

#### Holotype.

Specimen circled on slide DIA2025002 (= Fig. 7B), deposited in the Herbarium of Jishou University (JIU), China. Registration: http://phycobank.org/105637.

#### Type locality.

China. Qinghai Province, Lake Qinghai, a sampling site near the lakeshore, 36°50'34"N, 99°42'39"E, 3210 m asl., collected by Bing Liu, July 19^th^, 2019.

#### Description.

***LM*** (Fig. 7). Valve outlines ovate with broadly rounded headpole and cuneate footpole. Valve dimensions (n = 34): length range 38–57 μm, width range 31–50 μm at its widest region. Costa-stria bundles (CSBs) distinct, alternating with over-fibula ribs (OFRs) from pole to pole (labelled in Fig. 7E). CSBs radiate throughout valve surface. Fibulae visible, short (judged by OFRs, labelled in Fig. 7E), 4–6 in 10 μm. A marginal row of costae produced on the wall of raphe canal (e.g., Fig. 7B, arrows), 16–18 in 10 μm.

***SEM*** (Figs 8–11). Externally, raphe canal located directly on mantle (Fig. 8A, B), and wall of raphe canal bearing a marginal row of costae (Fig. 8B, arrows; Fig. 8E, double-headed arrows). Valve surface presents three differentiable areas (labelled A1, A2, and A3 in Fig. 8B). Distal raphe endings straight, interrupted at both headpole and footpole (Fig. 8C, D). Surface costae mostly slightly raised, not reaching valve midline except at two poles (Fig. 8A, B). Each CSB often composed of ca. 2–4 costae and 3–5 striae (Fig. 8F). Outside openings of areolae slit-like (Fig. 8D). Striae multiseriate (sometimes becoming uniseriate at valve middle, Fig. 9A, D), composed of ca. 2–5 rows of areolae (Figs 8D, 9, 10), 16–20 in 10 μm (measured at the valve margin from SEM images, n = 3). Surface siliceous warts and reticulate thickenings produced on costae and between adjacent two costae, respectively (Fig. 8 A-F). Each mantle sinking corresponding to each over-fibula rib (Fig. 9C, arrows). Internally, wall of raphe canal not growing conspicuously into cell cavity, leaving portulae visible (Fig. 10). Fibulae slim, much shorter, spanning ca. 1/8 of valve width, far away from valve midline except at two poles. Marginal trough-like depressions present around entire raphe canal (Fig. 10). Raphe continuous at headpole (Fig. 10C, arrow) whereas interrupted at footpole (Fig. 10B, two arrows). 2–4 portulae produced between two adjacent fibulae (Fig. 10E, F, arrows). Inner openings of areolae rounded, rimmed (Fig. 10B–F).

#### Etymology.

Named after Lake Qinghai, where this species was found.

#### Vernacular name.

The Chinese name is “青海湖双菱藻”.

#### Ecology and distribution.

*Surirella qinghainensis* was commonly found in the surface sediment on the surface of the stones collected in Lake Qinghai with *S. brightwellii* and *S. ectorii.* It is more frequent than *S. ectorii* but less than *S. brightwellii*. So far, *S. qinghainensis* has only been found in the type locality and can be considered a brackish diatom species endemic to Lake Qinghai.

## ﻿Discussion

It is nearly impossible to identify our specimens collected from Lake Qinghai as *Surirella brightwellii* if only consulting its original description by Smith (1853, table 1), because Smith’s description was very simple and only provided one line drawing. However, we can see the ovate valve outline and the distinct costa-stria bundles (CSBs) alternating with over-fibula ribs (OFRs) on the valve surface, especially the fibulae spanning ca. 1/4 of the width of the valve, based on the clear LM illustrations provided in the paper of Krammer & Lange-Bertalot (1987: figs 42–45). Our specimens agree with the population of *S. brightwellii* sensu Krammer and Lange-Bertalot (1987: figs 42–45), and both were found in brackish water. Thus, we identify our specimens (Figs 1–3) as *S. brightwellii*. The detailed structures of the important characters—fibulae, portulae, and marginal trough-like depressions—are clearly illustrated for the first time for *S. brightwellii* (Fig. 3), which provides a useful reference for the correct identification of this species.

In *Surirella*, the polarity of the valves is an important character for identification at the species level and even comes first in the dichotomous key of Krammer and Lange-Bertalot (1997). In some species, the two apices are identical (isopolar species), i.e., the valve is symmetric to the transversal axis, while in other species the two apices differ in shape. In these heteropolar species, the apices can be referred to as the headpole and footpole. *Surirella ectorii* sp. nov. is a heteropolar species, but its valve outline is nearly elliptical so that it can be mistaken for an isopolar species (Fig. 4). Species of *Surirella* with a valve outline similar to *S. ectorii* are rare. *Surirella sella* Hustedt, a fossil species from Chile, has a quasi-isopolar outline, but the apices are more broadly rounded than in *S. ectorii*. In addition, the interior of the valve, as illustrated in Rumrich et al. (2000), is characterized by long fibulae forming a strongly silicified rib system and is therefore very different from that of *S. ectorii*. *Surirella comis* A.W.F. Schmidt, a marine species, also possesses broadly elliptical valves with almost equal apices (Sims 1996), but its valve interior is also completely different from that of *S. ectorii*. In *S. comis*, the marginal costae are short, and the central area is structureless except for two longitudinal bands of short striae that enclose the axial area and do not reach the ends of the valve (Ognjanova-Rumenova and Buczkó 2015). Interestingly, in *Surirella ectorii*, a marginal row of costae is produced on the wall of the raphe canal (Fig. 5), a character never observed in other species of *Surirella* for which SEM illustrations are available.

The most similar species to *S. qinghainensis* are *S. crumena* and *S. peisonis*, and all of them are brackish-water species. A simple description of *S. crumena* was given by Kützing (1849), and more useful details were provided by Krammer and Lange-Bertalot (1987) and Jüttner et al. (2025, table 2). *Surirella qinghainensis* and *S. crumena* have similar valve outlines, overlapping valve dimensions, CSBs alternating with OFRs, and can be found in brackish habitats (Table 2). However, in *S. qinghainensis*, the footpole is more cuneate, and the fibulae are much shorter than in *S. crumena* (Table 2). A simple description of *S. peisonis* was provided by Pantocsek (1902), and more useful details were given by Krammer & Lange-Bertalot (1987, table 2). *Surirella qinghainensis* and *S. peisonis* again have similar valve outlines, CSBs alternating with OFRs, short fibulae, and live in similar habitats (Table 2), but the two species can be easily distinguished by their valve dimensions, which are much smaller in *S. qinghainensis* than in *S. peisonis* (Table 2).

**Table 2.** Character comparison among *Surirella* species included in this study.

Feature	S. ectorii sp. nov.	S. comis	S. sella	S. qinghainensis sp. nov.	S. crumena	S. peisonis	S. brightwellii	S. brightwellii
Reference	This paper	Ognjanova-Rumenova and Buczkó (2015)	Rumrich et al. (2000)	This paper	Kützing, F.T. (1849), Krammer and Lange-Bertalot (1987), Jüttner et al. (2025)	Pantocsek 1902, Krammer and Lange-Bertalot (1987)	This paper	W. Smith 1853, Krammer and Lange-Bertalot (1987)
Valve outline	Nearly elliptical	Nearly elliptical	Nearly elliptical	Triangle-ovate	Ovate	Ovate	Ovate	Ovate
Valve length (L) and width (W) (μm)	L: 44–69; W: 34–45	L: 95; W: 75	No data	L: 38–57; W: 31–50	L: 30–65; W: 27–31	L: 112–120; W: 58.5–60	L: 19–56; W: 13–29	L: .28–41; No data on width
Stria type and density	Multiseriate, composed of 2–5 rows of areolae, 15–19 in 10 μm	Uniseriate, 22–23 in 10 μm	Uniseriate, 22 in 10 μm	Multiseriate, composed of 2–5 rows of areolae, 16–20 in 10 μm	No data	No data	Multiseriate, composed of 2–5 rows of areolae, 19–22 in 10 μm	No data
Costa-stria bundles (CSBs) and its composition	Distinct, each CSB composed of 2–5 costae and 3–6 striae	Distinct, each CSB composed of 11–14 striae	Distinct, each CSB composed of 1–2 costae and 8–14 striae	Distinct, each CSB composed of 2–4 costae and 3–5 striae	Distinct	Distinct	Distinct, each CSB composed of 2–4 costae and 3–5 striae	Distinct
Over-fibula rib (OFR)	Distinct	Distinct	Distinct	Distinct	Distinct	Distinct	Distinct	Distinct
Fibulae and their density	Slim, short, spanning ca. 1/4 of valve width, 4–6 in 10 μm	Short, 1.5–2 in 10 μm	Slim, very long, 2–3 in 10 μm	Slim, short, spanning ca. 1/8 of valve width, 4–6 in 10 μm	Distinct, spanning ca. 1/4 valve width	Short, spanning ca. 1/8 of valve width	Slim, short, spanning ca. 1/4 of valve width, 5–7 in 10 μm	Short, spanning ca. 1/4 of valve width
Number of portulae	2–5	No data	1–2	2–4	No data	No data	2–5	No data
Internal raphe endings at two apices	Continuous at headpole, interrupted at footpole	No data	No data	Continuous at headpole, interrupted at footpole	No data	No data	Continuous at headpole, interrupted at footpole	No data
Marginal row of costae	Present, distinct	No data	Absent	Present, distinct	No data	No data	Absent	No data

With the two new species described in this study, we have reached 10 new taxa described from Lake Qinghai in the past 10 years or so (Table 1). In the future, we plan to carry out a more systematic analysis of the diatom flora of this lake. Most of the rivers that feed the lake (about 50) are located in the northwest. Five of these rivers—the Buha, Shaliu, Quanji, Ha-er-gai, and Heima—contribute about 80% of the inflow (Li et al. 2007). The locations where these freshwater rivers enter the brackish lake could represent particularly diverse habitats, with variable levels of salinity. They have so far never been investigated for diatoms and thus represent very promising sampling locations for future studies. This next step should allow us to better determine the level of endemism of the diatom flora of this ancient lake.

## Supplementary Material

XML Treatment for Surirella brightwellii

XML Treatment for Surirella ectorii

XML Treatment for Surirella qinghainensis

## ﻿Additional information

### Use of AI

No use of AI was reported.

### Author ORCIDs

Ya-Lun Ma https://orcid.org/0009-0003-7838-3494

Qiao-Mu Peng https://orcid.org/0009-0008-6243-3780

Patrick Rioual https://orcid.org/0000-0001-9491-9197

Bing Liu https://orcid.org/0000-0002-8516-325X

Ji-Yan Long https://orcid.org/0000-0001-8160-089X

Bin Yang https://orcid.org/0000-0002-1123-9477

### Data availability

All of the data that support the findings of this study are available in the main text.
